# Mass Screening for Primary Open-Angle Glaucoma: Is It Really Relevant?

**DOI:** 10.7759/cureus.69736

**Published:** 2024-09-19

**Authors:** Amine Razzak, Mohamed Bouazza, Loubna Mouhib, Mehdi Khamaily, Mohamed Elbelhadji

**Affiliations:** 1 Department of Ophthalmology, Cheikh Khalifa International University Hospital, Mohammed VI University of Sciences and Health, Casablanca, MAR; 2 Research Unit, Mohammed VI Center for Research and Innovation, Rabat, MAR

**Keywords:** blindness, glaucoma detection, glaucoma screening, mass screening, primary open-angle glaucoma

## Abstract

Glaucoma, one of the leading causes of blindness worldwide, poses a significant public health challenge. Despite advancements in diagnostic technologies, early detection remains a challenge. This study assesses the relevance of glaucoma screening in a population-based context.

Conducted in October 2023, this retrospective study involved 434 employees at two pharmaceutical laboratory sites. The average age was 44 years, with a female-to-male ratio of 0.7. A family history of glaucoma was reported in 74 participants (17%). Intraocular pressure (IOP) measurements showed that 20 participants (4.6%) had IOP above 21 mmHg, while the average IOP in the studied population was 15.5 mmHg. Glaucomatous damage to the optic nerve head was observed in 65 cases (15%).

A total of 71 employees (16.5%), including 70% women, were re-examined in a hospital setting, where they underwent comprehensive ophthalmological examinations. Among them, 13 participants (18%) were diagnosed with glaucoma, representing 2.9% of the initially screened employees.

This study highlights the ongoing challenge of preventing irreversible blindness due to glaucoma. Although the diagnostic approach used was effective for early detection among employees, the overall value of mass glaucoma screening remains debated. This debate is due to the disease's low prevalence, the lack of highly sensitive and specific screening tests, economic considerations, and the impact on the quality of life of those screened. Further studies are needed to evaluate the clinical effectiveness and economic efficiency of various screening strategies.

## Introduction

Primary open-angle glaucoma (POAG) is one of the leading causes of irreversible blindness worldwide. It is a chronic and progressive optic neuropathy that leads to the gradual loss of retinal nerve fibers and characteristic visual field deficits. It represents a significant public health issue [1].

Intraocular pressure (IOP) remains the primary risk factor for this disease and the only modifiable one, highlighting the importance of its measurement and control in managing this condition [2]. Despite advances in diagnostic technologies, early detection of POAG remains challenging due to the disease's often asymptomatic nature.

On an individual level, systematic screening for POAG enables early diagnosis of structural and functional alterations associated with the disease through a combination of various clinical and paraclinical diagnostic tests. However, the relevance of population-based screening for this condition remains controversial and raises many questions due to the lack of evidence regarding the clinical and economic efficacy of mass screening programs [3].

This study aims to evaluate the relevance of glaucoma screening in a population-based context by examining the results of a screening campaign.

## Materials and methods

This was a retrospective observational study conducted in October 2023 at two pharmaceutical production sites in Casablanca, where two glaucoma screening campaigns were organized for employees.

The study included individuals who were either over the age of 40 and/or had a family history of glaucoma. Those already diagnosed with glaucoma or who were lost to follow-up were excluded from participation.

A detailed questionnaire was administered to collect information on risk factors for POAG, such as melanoderma, myopia, family history of glaucoma, diabetes, corticosteroid use, smoking, and sleep apnea syndrome [4,5].

All participants in the two screening campaigns underwent IOP measurement three times using a portable air-puff tonometer (TonoCare®, Keeler, Windsor, UK), with 21 mmHg set as the upper limit of normality. Retinography using a portable non-mydriatic retinal camera (MicroClear®, Suzhou MicroClear Medical Instruments Co., Ltd., Suzhou, China) was performed on all participants, with qualitative and quantitative analysis of the optic disc to detect glaucomatous signs, such as a cup-to-disc ratio > 0.6, asymmetry of optic disc excavation between the two eyes, thinning of the neuroretinal rim, presence of a notch or disc hemorrhage, exclusion of a circumlinear vessel, and the presence of peripapillary atrophy.

The IOP measurements and retinography analyses were conducted by ophthalmology specialists. Individuals suspected of having POAG due to ocular hypertension and/or suspected glaucomatous optic disc changes were invited for a comprehensive ophthalmological examination at the Ophthalmology Department, which included several key evaluations. Visual acuity and refraction were first assessed, and then IOP correlated with pachymetry was measured using a Goldmann applanation tonometer (Haag-Streit, Köniz, Switzerland) and an optical pachymeter (Topcon®, Topcon Corporation, Tokyo, Japan). The anterior segment of the eye was examined with a slit lamp to check for signs of pseudo-exfoliation, pigment dispersion syndrome, or other indications of secondary glaucoma. A gonioscopy with a three-mirror contact lens was performed to exclude an angle-closure glaucoma. Additionally, automated visual field testing (24-2 Humphrey) was conducted, and if central visual damage was suspected, a more detailed 10-2 visual field test was included. Finally, optical coherence tomography (OCT) of the optic disc and macular ganglion cells (Topcon® 3D OCT-2000 version 8.11, Topcon Corporation, Tokyo, Japan) was carried out to provide detailed images and assess potential glaucomatous damage.

The diagnosis of POAG was confirmed based on several clinical (high IOP associated with progressive glaucomatous optic neuropathy) and paraclinical (deficit in peripheral visual field and damage to the retinal nerve fiber layer and macular ganglion cells on OCT) criteria, considering the presence of one or more risk factors.

## Results

Among the 434 employees included in this study, the average age was 44 years, with a range from 25 to 60 years. The female-to-male ratio was 0.7. Table 1 summarizes the risk factors for glaucoma identified in the studied population.

**Table 1:** Glaucoma risk factors

Risk factors	Number (N)	Percentage (%)
Family history of glaucoma	74	17%
Arterial hypertension	91	21%
Diabetes	55	12.7%
Chronic smoking	39	9%
Myopia	19	4.5%

IOP measurements revealed that 20 participants (4.6%) had IOP above 21 mmHg, with the average IOP being 15.5 mmHg and ranging from 10 to 31 mmHg. Examination of the optic nerve head revealed clinical signs of glaucomatous damage in 65 cases (15%).

A total of 71 employees (16.5%), including 70% women, were re-examined at our hospital facility, where they received a complete ophthalmological examination. Among these individuals, 39 (55%) underwent automated perimetry and OCT of the optic disc and macular ganglion cells. The average pachymetry among these individuals was 515 µm, ranging from 435 to 590 µm.

Of the 71 re-examined individuals, 13 (18%) were diagnosed with glaucoma at the end of the two screening campaigns, representing 2.9% of the initially screened employees (N = 434). Therapeutic management primarily involved medical treatment, with monotherapy in 10 cases (76%) and combination therapy in three cases (24%).

## Discussion

POAG is a chronic condition that remains asymptomatic for a long time and can lead to blindness due to its painless nature and late onset of central vision loss. Its prevalence varies by age and ethnicity; according to the Baltimore Eye Survey, it is 1-2% among individuals aged 40 to 49 years. For those over 70, it ranges from 2% to 3% in white individuals to 11% in Black individuals [6]. Bron et al. reported a prevalence of high-pressure glaucoma of 2.2% in men and 3% in women [7]. This study's prevalence of 2.9% is comparable to similar studies using different screening methods.

Several studies identify IOP level and advanced age as major risk factors for the disease [8], with additional factors including heredity, thin pachymetry, melanoderma, moderate to severe myopia, and diabetes [9]. This study included individuals over 40 to target the high-risk population, with a family history of glaucoma and diabetes being the main risk factors found.

On an individual level, early diagnosis and treatment of POAG can reduce disease progression risk through a combination of clinical and paraclinical tests [10]. However, mass screening for this condition raises several concerns despite numerous studies evaluating various glaucoma screening technologies. Few studies have assessed the economic cost and potential benefits of public health indicators and quality of life [11-15].

IOP measurement remains one of the most commonly used tools in glaucoma screening and diagnosis, though it is insufficient alone as it may lead to false negatives and/or positives [16]. In mass screening for POAG, tonometry alone has limited utility regardless of the technique used. No IOP threshold provides sufficient sensitivity and specificity for a reliable population-based screening test; thus, it should be combined with other screening methods, such as optic disc examination and perimetry [8,17].

Analysis of optic disc alterations, clinically or through retinography, remains a crucial tool in glaucoma screening despite considerable morphological variations among individuals, which affects its specificity and sensitivity [18]. Lamoureux et al. evaluated the performance of optic disc photographs taken with a non-mydriatic retinal camera, finding a sensitivity of 87.5% and specificity of 94% [19].

Other studies have evaluated the utility of functional tests such as automated perimetry or frequency doubling technology (FDT), either alone or combined with other clinical and/or paraclinical tests for mass screening. The sensitivity of FDT varies from 7% to 92%, depending on the study [20,21]. While promising and more effective when combined with other screening methods, its practical implementation remains challenging and costly, limiting its utility in population-based settings.

In our study, we opted for the use of a portable air tonometer coupled with a non-mydriatic retinograph, which combines several advantages due to its low cost and ease of use. Additionally, they are suitable for use in occupational health settings.

Population screening for glaucoma using a combination of several clinical and paraclinical tests seems to be an attractive methodological approach to detecting the early stages of the disease. However, no randomized study establishing the clinical and economic efficacy of these technologies and their impact on the quality of life of screened patients has been published [22-24].

According to the criteria established by the UK National Screening Committee regarding the relevance of establishing a population screening program, summarized in Table 2, the disease being screened must be an important public health issue. However, due to its low prevalence, no test has proven its superiority in terms of sensitivity and specificity to make it a reliable and reproducible screening test for POAG [25]. This was corroborated by our study, as only 2.9% of the screened individuals were declared glaucomatous at the end of the study despite the use of a rigorous diagnostic approach combining several clinical and paraclinical tests.

**Table 2:** Public health criteria justifying the establishment of a population screening program According to the UK National Screening Committee [25].

Disease’s characteristics	Criteria for disease screening
Public health importance	The disease must be a significant public health problem.
Disease understanding	The epidemiology and natural progression of the disease must be well known.
Early detection	Risk factors and the disease's latency period should be detectable at an early stage.
Benefits of early management	Early management must be significantly more beneficial than treatment at an advanced stage.
Screening test requirements	The test must be sensitive, specific, and acceptable to the population, and the intervals for repeating it must be defined.
Resource availability	Sufficient resources must be available to support the screening activity.
Risk-benefit ratio	The physical and psychological risks of screening must be lower compared to its benefits.
Cost-effectiveness	The benefits of population screening must justify its cost.

Moreover, the natural progression of the disease must be well understood, but this progression is not perfectly known in the case of POAG due to its great clinical heterogeneity. Indeed, no long-term prospective study on untreated patients has been conducted. Quigley estimated that in the absence of treatment, a Black person with glaucoma at the age of 40 would develop legal blindness around the age of 80 [26]. Screening would then mainly identify slowly progressing glaucoma and would not identify individuals with rapidly progressing glaucoma until the advanced stages of the disease.

Studies conducted in the UK and the US have concluded that mass screening is not economically viable because the prevalence of glaucoma remains relatively low, not justifying the cost required to conduct such screening programs. However, other studies should be conducted in low-income countries to evaluate the economic viability of such a program in a different socio-economic context [11,13,27].

POAG is a serious and chronic condition that affects visual function and the quality of life of patients. The impact of population screening on the quality of life of screened individuals is poorly evaluated in the literature, making it difficult to precisely determine the interest in establishing a screening program and its impact on quality of life [24,28].

The relevance of mass screening for glaucoma remains controversial due to its low prevalence and the absence of sufficiently sensitive and specific screening tests. Moreover, the economic aspects and the impact on the quality of life of screened patients remain poorly evaluated in the literature. The results of our study confirm the importance of traditional risk factors for POAG and highlight the effectiveness of the diagnostic approach adopted, particularly in an occupational health context. However, despite these encouraging results, our study has several limitations. First, the sample size was relatively small, which may impact the generalizability of the findings. Additionally, since the study was conducted within an industrial unit, the sample does not necessarily represent the epidemiological and socio-economic profile of our population, in which glaucoma diagnosis is often made at an advanced stage of the disease. Another limitation is that the use of IOP values as a diagnostic criterion does not account for cases of normal-tension glaucoma. Furthermore, economic and quality of life aspects were not included in this study.

## Conclusions

Combating irreversible blindness secondary to glaucoma remains a major challenge. Although our study demonstrated the effectiveness of the diagnostic approach used to detect glaucoma early in employees, the interest in mass glaucoma screening remains debatable. The challenges related to the low prevalence of the disease, the absence of sufficiently sensitive and specific screening tests, as well as the economic aspects and the impact on the quality of life of screened patients, mean that the conditions for mass screening are not currently met for POAG. As a result, only individual screening is currently of interest, underlining the need for additional studies to evaluate the clinical efficacy and economic efficiency of different screening strategies. Future research should also explore the interest in using new technologies, including telemedicine and artificial intelligence in mass glaucoma screening, to develop more accurate, cost-effective, and beneficial screening programs for public health.

## References

[1] Hu VH (2022). The global challenge of glaucoma. Community Eye Health.

[2] Gordon MO, Torri V, Miglior S (2007). Validated prediction model for the development of primary open-angle glaucoma in individuals with ocular hypertension. Ophthalmology.

[3] Harasymowycz P, Kamdeu Fansi A, Papamatheakis D (2005). Screening for primary open-angle glaucoma in the developed world: are we there yet?. Can J Ophthalmol.

[4] Bonovas S, Peponis V, Filioussi K (2004). Diabetes mellitus as a risk factor for primary open-angle glaucoma: a meta-analysis. Diabet Med.

[5] Le A, Mukesh BN, McCarty CA, Taylor HR (2003). Risk factors associated with the incidence of open-angle glaucoma: the visual impairment project. Invest Ophthalmol Vis Sci.

[6] Tielsch JM, Sommer A, Katz J, Royall RM, Quigley HA, Javitt J (1991). Racial variations in the prevalence of primary open-angle glaucoma. The Baltimore Eye Survey. JAMA.

[7] Bron A, Baudouin C, Nordmann JP (2006). Prevalence of intraocular hypertension and glaucoma in a nonselected French population. J Fr Ophtalmol.

[8] Kass MA, Heuer DK, Higginbotham EJ (2002). The Ocular Hypertension Treatment Study: a randomized trial determines that topical ocular hypotensive medication delays or prevents the onset of primary open-angle glaucoma. Arch Ophthalmol.

[9] Bron A, Chaine G, Villain M (2008). Risk factors for primary open-angle glaucoma. J Fr Ophtalmol.

[10] Heijl A, Leske MC, Bengtsson B, Hyman L, Bengtsson B, Hussein M (2002). Reduction of intraocular pressure and glaucoma progression: results from the Early Manifest Glaucoma Trial. Arch Ophthalmol.

[11] John D, Parikh R (2017). Cost-effectiveness and cost utility of community screening for glaucoma in urban India. Public Health.

[12] Mvoulana A, Kachouri R, Akil M (2019). Fully automated method for glaucoma screening using robust optic nerve head detection and unsupervised segmentation based cup-to-disc ratio computation in retinal fundus images. Comput Med Imaging Graph.

[13] Tang J, Liang Y, O'Neill C (2019). Cost-effectiveness and cost-utility of population-based glaucoma screening in China: a decision-analytic Markov model. Lancet Glob Health.

[14] Thau AJ, Rohn MC, Biron ME (2018). Depression and quality of life in a community-based glaucoma-screening project. Can J Ophthalmol.

[15] Einarson TR, Vicente C, Machado M, Covert D, Trope GE, Iskedjian M (2006). Screening for glaucoma in Canada: a systematic review of the literature. Can J Ophthalmol.

[16] Sommer A (1996). Glaucoma risk factors observed in the Baltimore Eye Survey. Curr Opin Ophthalmol.

[17] Detry-Morel M, Zeyen T, Kestelyn P, Collignon J, Goethals M (2004). Screening for glaucoma in a general population with the non-mydriatic fundus camera and the frequency doubling perimeter. Eur J Ophthalmol.

[18] Harper R, Reeves B (2000). The sensitivity and specificity of direct ophthalmoscopic optic disc assessment in screening for glaucoma: a multivariate analysis. Graefes Arch Clin Exp Ophthalmol.

[19] Lamoureux EL, Lo K, Ferraro JG, Constantinou M, Keeffe JE, Müller A, Taylor HR (2006). The agreement between the Heidelberg Retina Tomograph and a digital nonmydriatic retinal camera in assessing area cup-to-disc ratio. Invest Ophthalmol Vis Sci.

[20] Mansberger SL, Johnson CA, Cioffi GA (2005). Predictive value of frequency doubling technology perimetry for detecting glaucoma in a developing country. J Glaucoma.

[21] Yamada N, Chen PP, Mills RP, Leen MM, Lieberman MF, Stamper RL, Stanford DC (1999). Screening for glaucoma with frequency-doubling technology and Damato campimetry. Arch Ophthalmol.

[22] Robin TA, Müller A, Rait J, Keeffe JE, Taylor HR, Mukesh BN (2005). Performance of community-based glaucoma screening using Frequency Doubling Technology and Heidelberg Retinal Tomography. Ophthalmic Epidemiol.

[23] Sanchez-Galeana C, Bowd C, Blumenthal EZ (2001). Using optical imaging summary data to detect glaucoma. Ophthalmology.

[24] Fleming C, Whitlock EP, Beil T, Smit B, Harris RP (2005). Screening for primary open-angle glaucoma in the primary care setting: an update for the US preventive services task force. Ann Fam Med.

[25] Burr JM, Mowatt G, Hernandez R (2007). The clinical effectiveness and cost-effectiveness of screening for open angle glaucoma: a systematic review and economic evaluation. Health Technol Assess.

[26] Broman AT, Quigley HA, West SK (2008). Estimating the rate of progressive visual field damage in those with open-angle glaucoma, from cross-sectional data. Invest Ophthalmol Vis Sci.

[27] Burr J, Hernández R, Ramsay C (2014). Is it worthwhile to conduct a randomized controlled trial of glaucoma screening in the United Kingdom?. J Health Serv Res Policy.

[28] Aspberg J, Heijl A, Bengtsson B (2021). Screening for open-angle glaucoma and its effect on blindness. Am J Ophthalmol.

